# Case report: a man with untreated rheumatoid arthritis, cryoglobulinemic vasculitis, membranous nephropathy and pulmonary sarcoidosis

**DOI:** 10.1186/s12882-020-02161-5

**Published:** 2020-11-19

**Authors:** Qiyu Wang, Juan P. Ruiz, Peter D. Hart

**Affiliations:** 1 Internal Medicine Residency Program, Department of Medicine, Cook County Health, Chicago, IL USA; 2 Department of Nephrology, Cook County Health, Chicago, IL USA

**Keywords:** Case report, Nephrotic syndrome, Membranous nephropathy, Rheumatoid arthritis, Cryoglobulinemic vasculitis, Pulmonary sarcoidosis

## Abstract

**Background:**

Glomerular involvement in rheumatoid arthritis has been known to be associated with treatment side effects from medications and secondary amyloidosis. However, limited basic science and clinical studies have been performed to address the potential disease specific immune-mediated mechanisms causing secondary glomerular pathology, its various types of presentation, and the potential treatments.

**Case presentation:**

A 41-year-old man with chronic active rheumatoid arthritis presented with nephrotic syndrome and was found to have membranous nephropathy with eosinophilic intracapillary thrombi on renal biopsy. Proteinuria persisted despite complete withdrawal from non-steroidal anti-inflammatory drugs (NSAIDs) and disease-modifying anti-rheumatic drugs (DMARDs). Throughout the disease course, he developed cryoglobulinemic vasculitis and pulmonary sarcoidosis, both of which achieved clinical resolution with glucocorticoids. However, only partial improvement was observed in proteinuria with treatment of steroids and Rituximab.

**Conclusion:**

Our case presented a unique and complicated clinical phenotype of active rheumatoid arthritis, with clinical features of cryoglobulinemic vasculitis, histopathologic features of membranous and cryoglobulinemic nephropathy in the absence of DMARDs use, as well as pulmonary sarcoidosis. We speculate that there is a wider spectrum of glomerular disease in patients with untreated rheumatoid arthritis. In addition, the potential association between rheumatoid arthritis and cryoglobulinemic vasculitis should probably be revisited and requires further studies to elucidate the underlying mechanisms and treatment options.

## Background

Rheumatoid arthritis (RA) is a chronic inflammatory condition that primarily affects the joints, with multi-system involvement including skin, heart and lungs. Renal manifestations, however, appear to be much less prevalent in the pre-existing literature. In the pre-methotrexate era, secondary amyloidosis was reported to be a major cause of chronic kidney disease (CKD) in patients with RA who had a prolonged disease course and high inflammatory markers. Long-term use of the nonsteroidal anti-inflammatory drug (NSAIDs) and traditional disease-modifying anti-rheumatic drugs (DMARDs) including penicillamine and gold are known to be responsible for most types of glomerular disease in patients with RA [1]. Other than glomerulonephritis secondary to chronic medication use, glomerular pathology has rarely been reported to be from a direct immune-mediated mechanism in patients with RA.

## Case presentation

A 41-year-old Hispanic man with history of rheumatoid arthritis (RA) and type2 diabetes mellitus was referred to the emergency room (ER) of a large urban city hospital from the rheumatology clinic for newly developed bilateral lower extremity rashes and edema after 1 year of being lost to follow-up. He was diagnosed with RA 2 years prior to admission after presenting with polyarthritis and strongly positive serum immunologic markers (rheumatoid factor titer 1220 IU/ml [normal range, < 20 IU/ml], anti-cyclic citrullinated peptide titer 240.05 U/ml [normal range, < 20 U/ml; strongly positive, > 60 U/ml]). Methotrexate, low dose prednisone and sulfasalazine had resulted in better control of disease activity. Unfortunately, the patient was lost to follow-up. He had been off of disease-modifying antirheumatic drugs (DMARDs) for RA and was only taking ibuprofen 800 mg every 8 h as needed for about a month for joint pain.

When the patient was seen in the ER (day 0), the physical exam showed pinpoint, non-tender, non-blanchable purpuric macules coalescing into large patches on the left leg, with smaller areas of involvement on the right leg. The skin lesions were in a dependent distribution involving more of the flexor surface than extensor surface. Joint exam revealed polyarticular arthritis with pain and swelling in the right 2nd and 3rd metacarpophalangeal (MCP) joints and left 3rd (MCP). Boutonniere deformities were observed in both hands (left more than right), as well as subcutaneous nodules under the elbow. Significant pitting edema was found in the lower extremities. The rest of the physical exam was unremarkable.

His urinalysis showed red blood cell of 21/high power field (hpf) and white blood cell 7/hpf. His creatinine was 0.9 mg/dl, with estimated GFR of 93 ml/min using MDRD equation. The diagnosis of nephrotic syndrome was established given large proteinuria (11 g/gm) on the spot urine sample, hypoalbuminemia (2.2 g/dL), and peripheral edema.

Further serology work-up showed low C3 of 86 (normal range 88–201), C4 of 24 (normal range 16–47), positive cryoglobulin qualitative with < 1% Cryocrit, as well as negative c-ANCA, p-ANCA, and ANA (including Anti-dsDNA, Anti-Smith, Anti-SSA and Anti-SSB). He had a polyclonal elevation in IgG (1790 mg/dl, normal range 694-1618 mg/dl) and IgA (661 mg/dl, normal range 68-378 mg/dl), with a normal IgM (181 mg/dl, normal range 77-220 mg/dl). There was absence of M-spike on serum protein electrophoresis. HIV, Hepatitis C and hepatitis B serologies were negative. Unfortunately, rheumatoid factor (RF) titer was not checked at that point of time. Prednisone was started at 10 mg daily empirically for treatment of nephrotic syndrome and active RA, and patient was referred to renal clinic for further diagnosis and monitoring of therapy.

Three days after being discharged (Day 5) and prior to being seen in the renal clinic, the patient developed painful purplish discoloration of the distal right thumb, which prompted another ER visit (Day 12). On exam, the distal phalanx was cool to touch and exquisitely tender on palpation with signs of onycholysis. There was no sign of cutaneous necrosis or surrounding cellulitis. Doppler ultrasound showed good radial and ulnar pulses with good blood flow to the distal phalanx of the right thumb. He was discharged from the ER since no macrovascular signs were found, and he was given follow up with rheumatology. On Day 16, rheumatology noticed new lower lip purpuric lesions, which, in conjunction to the positive cryoglobulin qualitative test along with the thumb and lower leg lesions, prompted an increase in Prednisone dose to 80 mg daily for treatment of systemic cryoglobulinemic vasculitis. Unfortunately, his skin lesions were not biopsied.

**Fig. 1:**
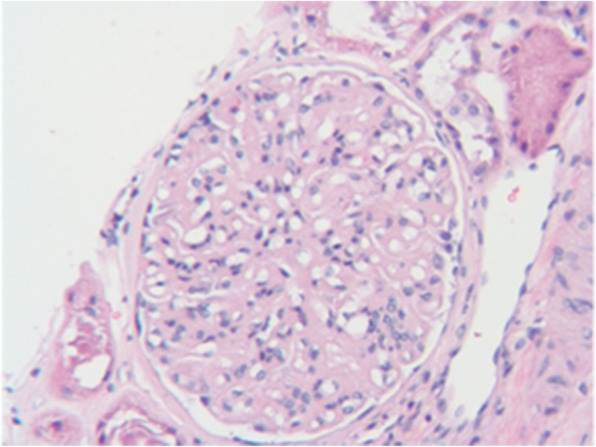
H&E, diffuse capillary wall thickening

**Fig. 2:**
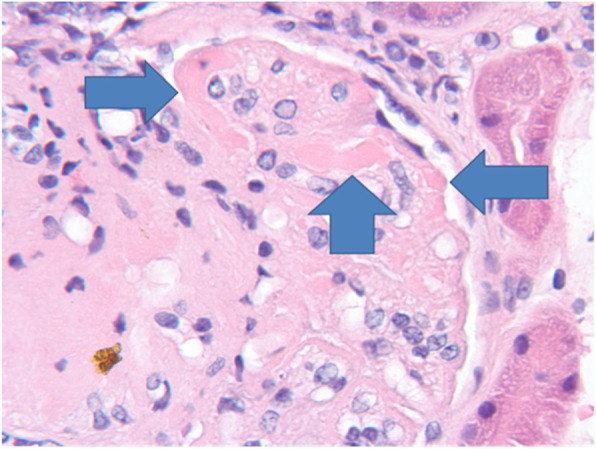
H&E, intracapillary thrombi (arrows)

**Fig. 3:**
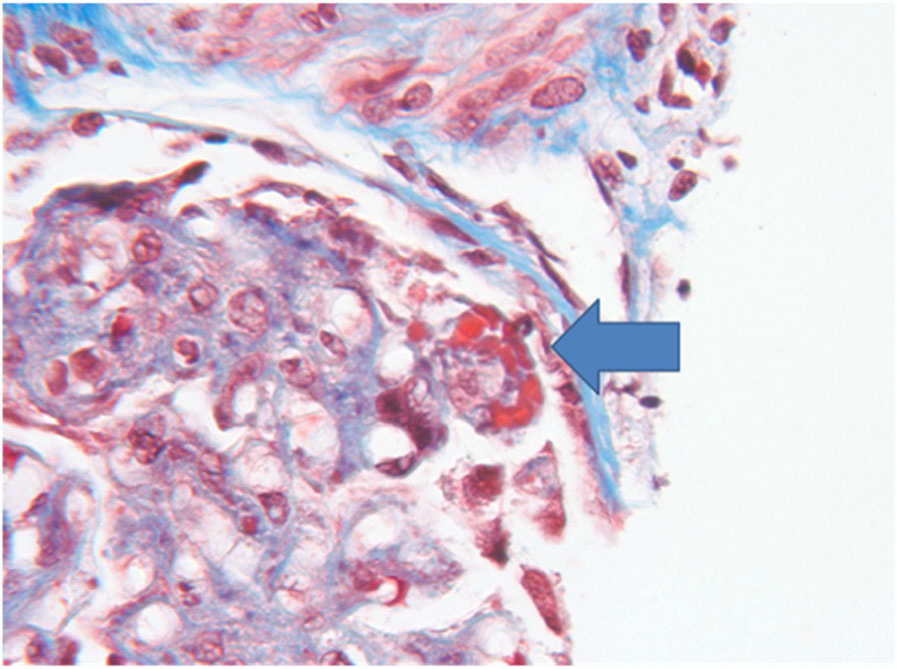
Trichrome stain, intracapillary thrombi, bright red (arrow)

**Fig. 4:**
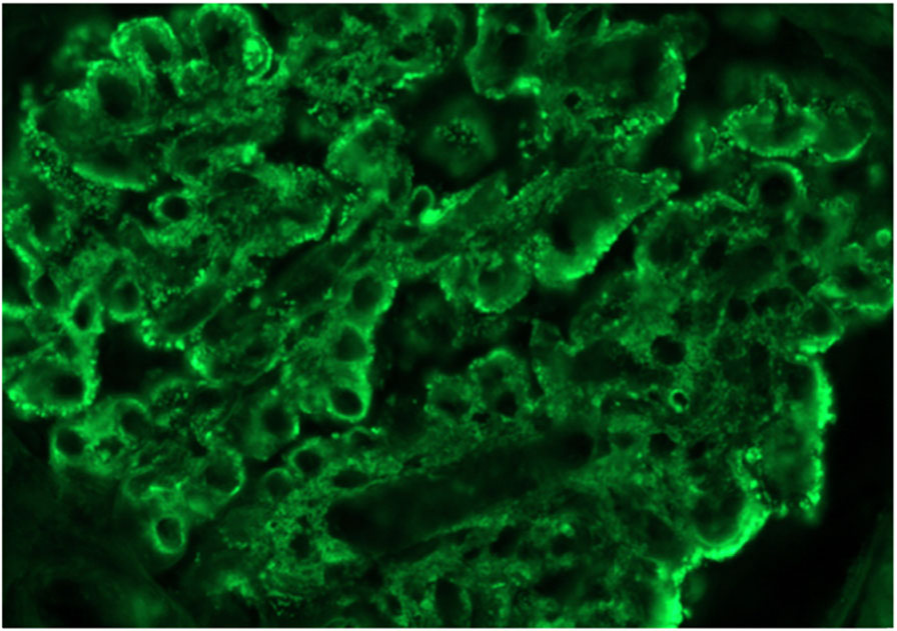
Immunofluorescence, diffuse granular staining for IgG

**Fig. 5:**
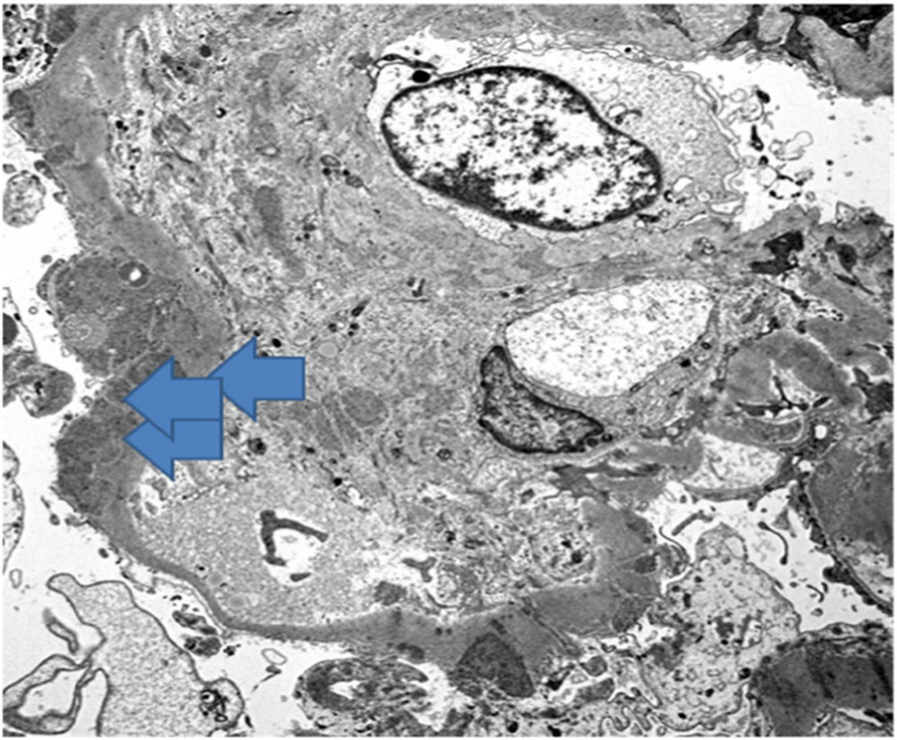
Electron microscopy, subepithelial deposits

Renal biopsy was performed on day 28. A total of 23 glomeruli were obtained, of which 1 was sclerosed. Light microscopy showed diffuse capillary wall thickening (Fig. 1) with rare eosinophilic intracapillary thrombi (Figs. 2, 3). Silver stain showed spikes and holes in the glomerular basement membrane. Immunofluorescence (IF) staining showed diffuse, finely granular deposits of IgG (Fig. 4), IgA, IgM, κ and γ light chains, C1q and C3. Anti-phospholipase A2 receptor (PLA2R) stain was negative. Electron microscopy (EM) showed widespread subepithelial and intramembranous electron-dense deposits, with diffuse foot process effacement (Fig. 5). Subendothelial or mesangial tubuloreticular inclusions suggestive of lupus nephritis were not found. The final pathologic diagnosis was compatible with membranous nephropathy with features of cryoglobulinemic glomerulonephritis. There were no signs of diabetic nephropathy on light microscopy.

Given the multisystem involvement (purpuric, non-blanching lesions in multiple skin and mucosal surfaces), characteristic pathological features (intracapillary thrombi) on renal biopsy and positive cryoglobulin qualitative test, a decision was made to start treatment empirically as systemic cryoglobulinemic vasculitis with Rituximab, considering that this is the first line treatment for hepatitis C-related cryoglobulinemic disease and evidence is limited for treatment of renal involvement in non-infectious mixed cryoglobulinemia. Unfortunately, treatment was delayed due to indeterminate Quantiferon and PPD tests.

One and a half months after starting high dose steroids (day 62), his right thumb and lower lip lesions had resolved, and his urine protein/creatinine ratio had decreased to 3.4 g/g.

Patient was started on Rituximab 1 g every 2 weeks and received 2 doses on day 67 and day 88, resulting in improvement of his proteinuria to 1.3 g/g (day 88). By this time, patient was on Prednisone 40 mg daily. Due to the persistently elevated blood sugar and Cushingoid features, a taper of the steroids occurred over the following 2 months.

Two months after he completed rituximab, the patient was admitted for presumed pneumonia (day 158) secondary to immunosuppression after presenting with 2-week history of dyspnea and cough. He underwent a CT scan of the chest with contrast, which showed hilar and mediastinal adenopathy without parenchymal consolidations. He underwent bronchoscopy with endobronchial ultrasound (EBUS), and the transbronchial lymph node biopsy of the mediastinal lymph node showed non-caseating granulomas. Acid fast stain and mycobacterium culture were negative. To rule out a lymphoproliferative disorder as a differential of non-caseating granulomas, PET scan, bone marrow biopsy and flow cytometry were performed, and they were negative for clonal lymphocytic proliferation. A clinical diagnosis of sarcoidosis was made, and his Prednisone dose was increased to 40 mg daily (patient was on a steroid taper and taking Prednisone 20 mg daily prior to this admission), resulting in rapid pulmonary symptoms improvement. No extrapulmonary signs of sarcoidosis developed in the course of his illness (e.g. erythema nodosum).

**Fig. 6:**
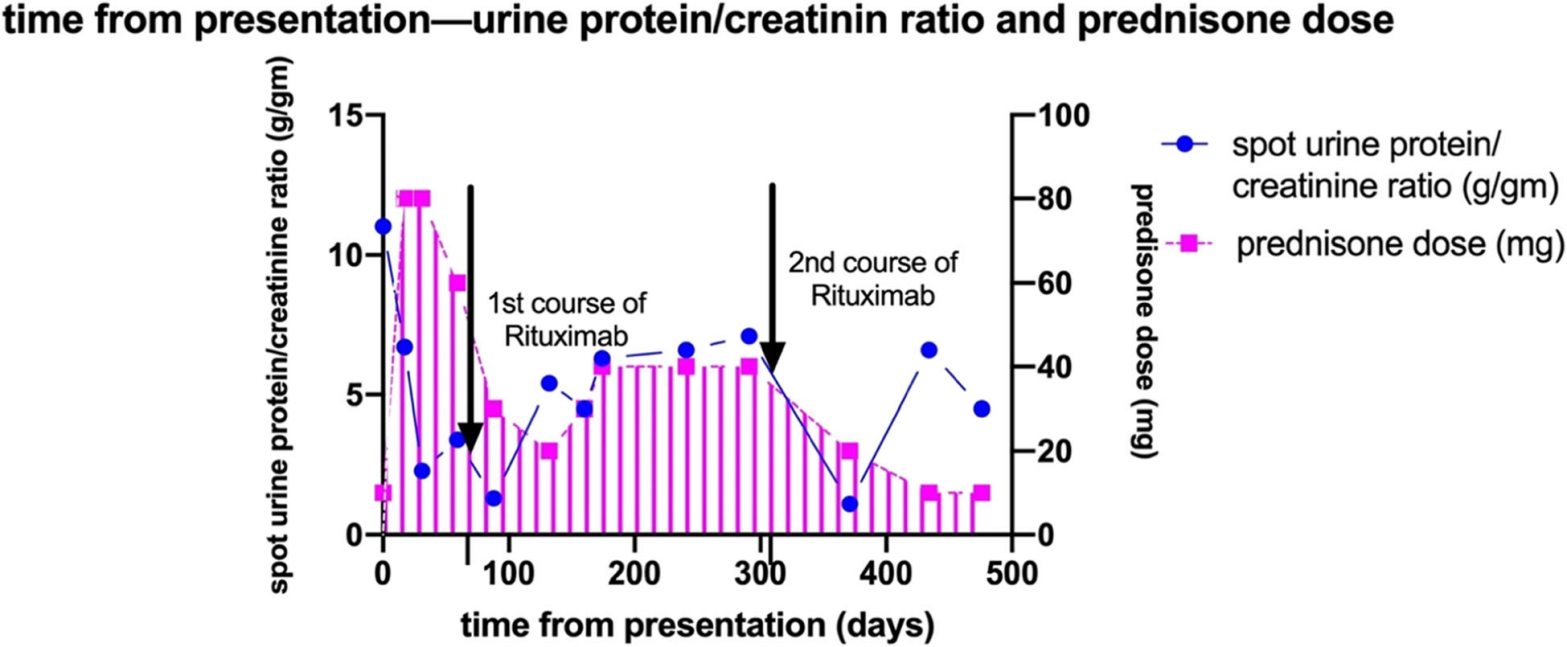
Horizontal line—days from the time of diagnosis; Vertical axis (left)—spot urine protein to creatinine ratio; Vertical axis (right)—prednisone dose; black arrows: first course of Rituximab treatment (day67 1000 mg, day 88 1000 mg) and second course of Rituximab treatment (day308 750 mg, day318 750 mg, day 325 750 mg, day 332 750 mg), respectively

**Fig. 7:**
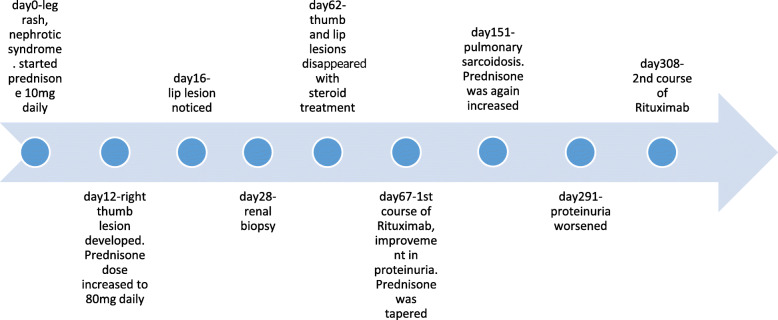
Timeline of clinical manifestation and diagnosis

Six months after his first course of Rituximab, his proteinuria had increased back to 7 g/gm (day 291), so a decision to repeat Rituximab course was made. Unfortunately, this was again delayed due to patient’s personal issues, and he started it 2 months later. This time he received weekly Rituximab 750 mg for a total of 4 doses. Patient again manifested a dramatic initial response to the treatment with reduction of proteinuria to 1.1 g/g (day 371), but with a slow increase in his urine protein to creatinine ratio over the following 3 months, to an average of 3.8 g/g, compatible with an overall partial response if compared with his initial proteinuria of 11 g/g on presentation (Fig. 6). His Creatinine level has remained stable between 0.8 ~ 1.1 mg/dl during the period of one and half year follow-up. His skin lesions and articular manifestations have completely resolved since the initiation of high dose steroids on day 62. Repeat Cryoprecipitate levels has been persistently positive. A timeline of the disease course is summarized in Fig. 7.

## Discussion and conclusions

We present a complicated case of membranous nephropathy, systemic cryoglobulinemic vasculitis with renal involvement, and pulmonary sarcoidosis in a patient with underlying chronic active rheumatoid arthritis. The patient experienced complete resolution of the cutaneous findings and pulmonary symptoms with high dose steroids, with partial improvement of the proteinuria after 2 courses of Rituximab therapy. We will discuss the diagnostic challenge of this case and the association of RA with this patient’s renal, cutaneous and pulmonary findings.

### Renal involvement in rheumatoid arthritis

Rheumatoid arthritis is a chronic inflammatory condition that primarily affects the joints, with multi-system involvement including skin, heart and lung. Renal manifestations, however, appear to be much less prevalent. In the pre-methotrexate era, secondary amyloidosis was reported to be a major cause of chronic kidney disease (CKD) in patients with RA who had a prolonged disease course (mean duration from the time of diagnosis 17.2 ± 7.3 years) and high inflammatory markers [2]. However, in recent years when DMARDs have been widely used, studies have suggested a weaker than previously thought association of RA disease duration and severity with the development of CKD. For example, one cross-sectional study performed in Japanese population showed that the strongest risk factors for CKD (defined by eGFR< 60 ml/hr) in patients with rheumatoid arthritis were hypertension and advanced age (> = 65-year-old), whereas the duration and disease activity of rheumatoid arthritis had a weaker association with the presence of CKD [3].

Long-term use of nonsteroidal anti-inflammatory drug (NSAIDs) and traditional disease-modifying anti-rheumatic drugs (DMARDs) including Penicillamine and gold are known to be the cause of most types of glomerular disease in patient with RA, with mesangial proliferative glomerulonephritis and membranous nephropathy being the most common pathological findings [1]. Other than secondary to chronic medication use, glomerular pathology has rarely been reported to be from a direct immune-mediated mechanism in patients with RA. In our patient, NSAIDs-induced secondary membranous nephropathy was deemed to be unlikely given the persistence of nephrotic-range proteinuria after complete withdrawal of ibuprofen for more than 5 months [4].

### The diagnosis of cryoglobulinemic vasculitis

According to the Brouet classification, type1 cryoglobulinemia involves monoclonal IgM and is typically associated with B cell lymphoproliferative diseases including Waldenstroms macroglobulinemia and multiple myeloma. Type 2 cryoglobulinemia involves monoclonal IgM against the Fc portion of polyclonal IgG, and is most commonly seen in chronic viral infections and systemic autoimmune diseases [5]. Chronic hepatitis C infection constituted 60 ~ 90% of the cases in type 2 mixed cryoglobulinemia in a large retrospective study in France [6], with Sjogren syndrome being the most common associated autoimmune condition. Type 3 cryoglobulinemia, however, is the least well described entity. It is suggested as a transient state between polyclonal gammopathy and type 2 cryoglobulinemia [5].

Similar to most other autoimmune conditions, “cryoglobulinemic vasculitis” lacks straightforward diagnostic criteria. In 2012, the Italian Study Group on Cryoglobulinemia (GISC) proposed a preliminary classification criterion for the cryoglobulinemic vasculitis, which includes fulfilling two out of the three set of items (clinical questionnaire, clinic presentation, and laboratory tests). This classification criteria provided a specificity of 95.4% with a reasonably good sensitivity 88.5% [7]. In our patient, his clinical manifestation fulfills the clinical questionnaire (episodes of red rashes on the skin and mucosal surface) and clinical presentation items (fatigue, purpura) with positive serum cryoglobulin tests 6 month apart, and should be considered as a likely diagnosis of cryoglobulinemic vasculitis. Our patient did not have any complaint of sensory or motor deficits during the clinical encounters, however, comprehensive neurological exam was not performed due to limited time of clinical encounter.

### Renal involvement in cryoglobulinemic vasculitis

Renal pathology was not included during the development of the aforementioned GISC criteria. Membranoproliferative glomerulonephritis (MPGN) was known to be the predominant histologic pattern in cryoglobulinemic vasculitis. In a retrospective analysis of the 62 cases of non-infectious mixed cryoglobulinemic glomerulonephritis (GN) identified in the French CryoVas Survey, 92.5% of the 62 cases had a pathological feature of MPGN, with mesangial proliferative GN constituting the rest of the pathology and none of the cases showing a membranous pattern on biopsy [6]. On detailed review of the renal pathology of our patient, there were rare eosinophilic intracapillary thrombi (Figs. 2, 3), which have been traditionally viewed as a characteristic feature of cryoglobulinemic GN, correlating with rapid immunoglobulin (often IgM) deposition into the glomerular basement membrane [8]. However, the specificity of this feature is less clear in the background of membranous GN, instead of MPGN.

### Diagnostic dilemma

The constellation of cutaneous vasculitis, positive serum qualitative cryoprecipitate and eosinophilic intracapillary thrombi representing immune globulin plugs in the glomerular tufts suggests an immune complex-mediated disease process—systemic cryoglobulinemic vasculitis. However, the glomerular pathology was incongruent with the pre-existing literature, which suggests that cryoglobulinemic glomerulonephritis rarely presents with membranous nephropathy. The immunofluorescent stain of our patient’s renal biopsy showed diffuse finely granular deposits of IgG (3+), IgA (3+), IgM (3+) with positive C3 and C1Q (or a “full house” pattern) typically seen in cryoglobulinemic glomerulonephritis. No macrophages were found in the intracapillary hyaline thrombi in the sampled glomeruli. It is also worth noticing that our patient does not have significant impairment of his GFR during the one and half year follow-up, with an overall Cr level ranging from 0.8 ~ 1.1 mg/dl (GFR 73 ~ 106 ml/min per MDRD equation). Reduced GFR is typically expected in patients with cryoglobulinemic vasculitis due to endocapillary proliferative lesions. Based on the aforementioned French CryoVas Survey, a mean GFR of 39.5 ml/min was observed in the cases of biopsy-proven cryoglobulinemic glomerulonephritis [6]. In addition, despite the “full-house” staining on immunofluorescence, lupus nephritis was deemed to be unlikely given a repeatedly negative serological profile, the lack of typical extra-renal lupus manifestations (e.g. malar rash, photosensitivity, oral ulcers), and lack of typical pathological features on renal biopsy (no signs of extra-glomerular immune deposits on IF, no signs of subendothelial or mesangial tubulo-reticular inclusions on electron microscopy).

**Fig. 8:**
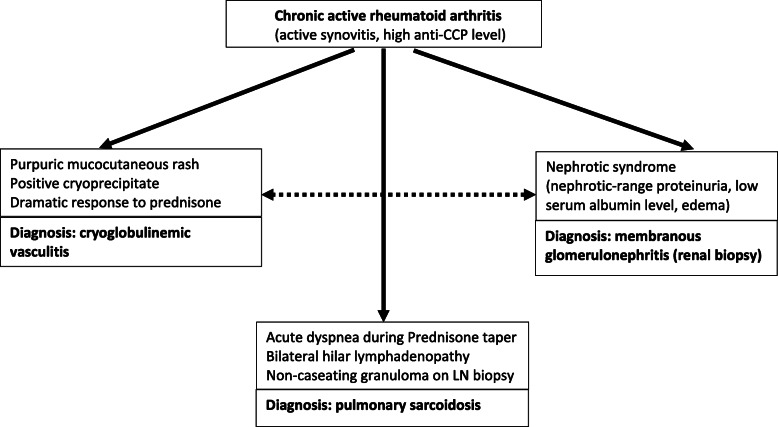
Diagnostic path

An alternative explanation of this complicated presentation is to split the cutaneous and renal findings: patient developed cryoglobulinemic vasculitis, while his membranous nephropathy being a separate co-existing disease process not directly related to the pathophysiology of cryoglobulinemic vasculitis. Primary membranous nephropathy appears to be unlikely given the negative serum level and staining for anti-phospholipase A2 receptor (PLA2R) in the renal biopsy. After excluding all known secondary causes of membranous GN, we propose that the glomerular pathology in our patient might be directly related to his underlying active RA probably through an immune-mediated mechanism, and it could potentially be part of the wider spectrum of glomerular disease in RA (Fig. 8). Cases of membranous nephropathy without other identifiable secondary causes as well as negative work-up for primary membranous GN have been reported in patients with long-standing rheumatoid arthritis [9].

### The association between rheumatoid arthritis and cryoglobulinemic vasculitis

The presence of serum cryoglobulin and vasculitis was described in early literature by Weisman et al. in 1975 as an associated condition in patients with RA. When comparing the 8 patients who had clinical evidence of vasculitis with those who did not, those who had vasculitis had a significantly higher RF titer and lower C3 level [10]. The term rheumatoid vasculitis was used to describe a type of necrotizing vasculitis that occurs in long-standing active rheumatoid arthritis. Interestingly, however, renal involvement was not present in any of the patients in the above study. The incidence of “rheumatoid vasculitis” and the use of this term has declined dramatically since the 1980s, which was attributed to better control of the inflammation with the introduction of multiple disease modifying agents and biologics [11].

Very few studies have described the association between rheumatoid arthritis and cryoglobulinemic vasculitis. In a retrospective analysis in patient with non-infectious type2 mixed cryoglobulinemia in Northern France, underlying RA was found in only 2 out of 33 patients studied, with Sjogren syndrome being the most common associated autoimmune condition [12]. In contrast to the relatively well-studied HCV-related mixed cryoglobulinemic vasculitis, the evidence of RA as the etiology of systemic cryoglobulinemic vasculitis has not been well established, and the potential mechanism has also not been elucidated. Given the biochemical plausibility that rheumatoid factor, an IgM that has affinity towards the Fc portion of IgG, forms large immune complexes which could potentially activate complement, an immune-complex mediated mechanism of the vasculitis was proposed.

### Treatment challenge

Despite the diagnostic difficulty, the decision was made to treat the patient with Rituximab, considering that it is increasingly considered as an effective treatment for primary membranous nephropathy, and is now preferred for the treatment of hepatitis C-associated cryoglobulinemic vasculitis over cyclophosphamide, in both instances. Unfortunately, limited evidence exists for the effectiveness of Rituximab and corticosteroid in patients with non-infectious mixed cryoglobulinemia. The patient showed significant treatment response immediately after treatment, however, his proteinuria quickly rebounded over a 2-month period (Fig. 6). A repeat renal biopsy was considered to evaluate for potential changes of pathology type (e.g. new signs of diabetic nephropathy), or persistent or worsening features of cryoglobulinemia which might raise a consideration of therapy switch to cyclophosphamide. However, due to the current COVID-19 pandemic and patient’s personal issues, the procedure has not been able to be arranged.

### The association between rheumatoid arthritis and sarcoidosis

Another interesting clinical manifestation during our patient’s disease course is the development of pulmonary symptoms which led to the diagnosis of pulmonary sarcoidosis. The patient developed cough and dyspnea after his first course of Rituximab treatment and while being quickly tapered off of Prednisone (patient was on Prednisone 20 mg daily when he developed clinical symptoms). The diagnosis of pulmonary sarcoidosis was established by consistent clinical, radiographical (bilateral hilar and mediastinal lymphadenopathy) as well as pathological evidence of non-caseating granuloma on mediastinal lymph node biopsy. The timing of this presentation is certainly intriguing in his complicated clinical course. On retrospective review of his prior chest radiographs obtained serially since his diagnosis, the hilar lymphadenopathy had been overall stable and didn’t show any significant changes at the time when the patient became symptomatic. His respiratory symptoms quickly resolved after up titrating his Prednisone dose to 40 mg daily. One possible explanation is that the patient had a symptomatic flare of pulmonary sarcoidosis during the rapid steroid taper, and his disease had remained asymptomatic clinically much longer prior the diagnosis of pulmonary sarcoidosis was made. Supportive evidence includes one of the observational studies performed in patients with pulmonary sarcoidosis who were treated with Prednisone, and who were observed clinically without initiation of therapy. Significant higher relapse rate was observed after clinical remission was achieved in patients who have received induction therapy with corticosteroids (74% vs. 8%, *p* < 0.01) [13].

Sarcoidosis has long been reported to co-exist with or mimic other autoimmune conditions including rheumatoid arthritis, suggesting overlapping immune dysregulation among these conditions. The clinical presentation of sarcoidosis has been reported to precede or lag behind the diagnosis of RA, and in some cases, the two conditions can manifest themselves at the same time [14]. Rituximab was also reported to be an effective treatment in some cases of extrapulmonary sarcoidosis, and is being studied as treatment for refractory pulmonary sarcoidosis [15]. More interestingly, with the introduction of biologics, cases of sarcoidosis being induced by the use of tumor necrosis factor alpha (TNF-α,) inhibitors (mostly etanercept) as treatment for RA have been reported over the past 20 years [16]. The exact pathophysiology of worsening of the granulomatous disease after introduction of biologics remains unexplained as TNF-α inhibitors would be expected to block the effect of TNF-α: an important cytokine responsible for macrophage activation and granuloma formation.

In summary, our case presented a unique and complicated clinical phenotype of active rheumatoid arthritis, with clinical features of cryoglobulinemic vasculitis, histopathologic features of membranous nephropathy in the absence of DMARDs use, and pulmonary sarcoidosis. All the clinical features showed an excellent response to corticosteroids, except for his renal manifestation, which only showed partial response to steroids and B-cell depletion therapy. We speculate that there is a wider spectrum of glomerular disease in patients with untreated RA. In addition, the association between rheumatoid arthritis and cryoglobulinemic vasculitis should be revisited and requires further studies to elucidate the underlying mechanisms and treatment options.

## Data Availability

Most of the clinical data used and analyzed in this case report is presented in this manuscript. More detailed information is available from the corresponding author on reasonable request.

## Abbreviations

RA: Rheumatoid arthritis
CKD: Chronic kidney disease
NSAIDs: Nonsteroidal anti-inflammatory drugs
DMARDs: Disease-modifying anti-rheumatic drugs
ER: Emergency room
MCP: Metacarpophalangeal
PIP: Proximal interphalangeal
RF: Rheumatoid factor
ACEI: Angiotensin converting enzyme inhibitor
PLA2R: Anti-phospholipase A2 receptor
EBUS: Endobronchial ultrasound
MPGN: Membranoproliferative glomerulonephritis
GN: Glomerulonephritis
